# Endocardiosis and congestive heart failure in a captive ostrich (*Struthio camelus*)

**Published:** 2013-11-09

**Authors:** M.A.G. Kubba, S.A. Al-Azreg

**Affiliations:** *Department of pathology, Faculty of Veterinary Medicine, University of Tripoli, Tripoli, Libya*

**Keywords:** Congestive heart failure, Endocardiosis, Myomatous valvular degeneration, Ostrich

## Abstract

A seven-year-old blue-necked male ostrich was found dead after a few days of illness. The animal was living in an open yard of 25 square meters along with three other females. They were given concentrate-rich ration with free access to green leaves and water. Autopsy revealed cardiac enlargement due to left ventricular hypertrophy and right ventricular dilatation. The left aterioventricular valves were irregularly thickened and contracted. The lungs were engorged with blood and the liver had nutmeg appearance. The small intestine showed segmental sub-serosal petechial hemorrhages. Histological examination revealed myxomatous degeneration of the left aterioventricular valves, pulmonary congestion and edema, congestion of periacinar hepatic zone and fatty degeneration of outer zones, renal glomerulosclerosis and arteriosclerosis. The affected parts of the small intestine showed villous atrophy with lacteal distention. The venules in the affected intestinal segment were severely dilated while the arterioles had narrow lumen and irregular wall thickening with hyaline deposition. The current article reports an endocardiosis in ostrich and discusses other vascular disorders.

## Introduction

Endocardiosis is a well-known cardiac disease, which affects certain breeds of dogs. It is a myxomatous degeneration of the valves, which mainly involves the left aterio-ventricular valves leading to malformation and mitral insufficiency.

Other cardiac valves are also affected, but with less severity and frequency. The affected valve is shortened and thickened, opaque white with smooth surface and no signs of inflammation. Microscopically, the most prominent features of endocardiosis are thickening of the valvular spongiosa and degeneration of the fibrosa.

The thickening of the former is due to proliferation of loose fibroblastic tissue rich with proteoglycans, hyaluronic acid and chondroitin sulfate (Jones *et al.*,1997; Jubb *et al.*, 2008; Kittleson, 2011; Fox, 2012). The cause of endocardiosis is not known, but is likely a genetically-influenced degeneration of connective tissue.

Though infrequent, valvular abnormalities suggestive of endocardiosis have also been reported in cats (Sisson *et al.*, 1999), horses (Reef *et al.*, 1998), pigs (Castagnaro *et al.*, 1997), Gambian rats (Muller *et al.*, 2010), rhesus monkeys (Schmidt, 1970) and in aviary birds (Schmidt *et al.*, 2008). This condition bears some resemblance to a condition in human referred to as mitral valve prolapse (Jones *et al.*, 1997). The current paper describes and discusses a condition in a captive ostrich, which corresponds well with endocardiosis in other animals.

### Case Details

Post mortem examination was conducted on a seven-year-old male ostrich, which has died after two days of illness. Specimens collected from the lung, heart, liver, intestine, kidney, trachea and testes were fixed in 10% neutral buffered formalin (NBF), dehydrated in rising concentrations of Ethyl alcohol, cleared in Xylol and embedded in paraffin wax. Sections of 5-6 microns were stained with Hematoxylin and Eosin (H&E). Alcian blue was used to stain mucopoly-saccharides in the affected valves and Crystal violet for detection of amyloidosis in the arterioles of the intestine and myocardium.

**Fig. 1:**
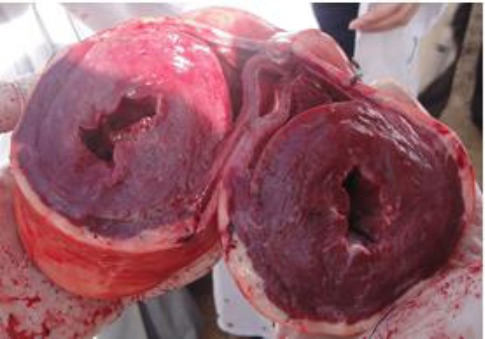
Heart: Left ventricular hypertrophy and right ventricular dilatation.

The main necropsy findings included cardiac enlargement due to left ventricular hypertrophy and right ventricular dilatation (Fig. 1).

**Fig. 2:**
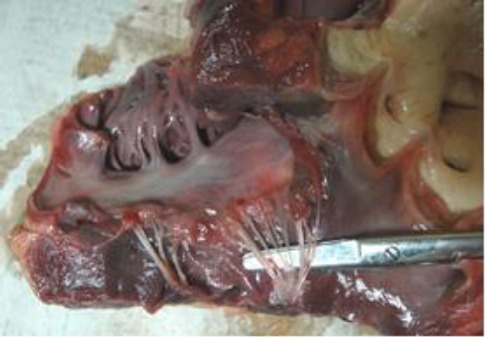
Mitral valve leaflets are fleshy, shrunk and nodular.

The left ventricle had thick muscular wall, prominent papillary muscles and reduced lumen. The mitral valve leaflets were unevenly thickened, fleshy and shrunk (Fig. 2).

**Fig. 3:**
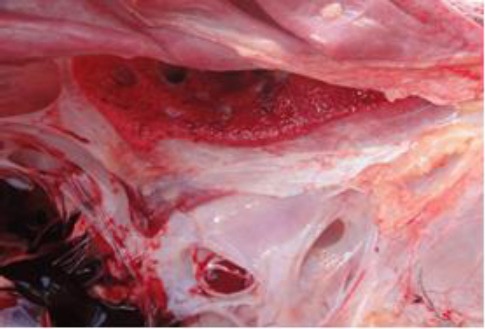
The lung is severely congested, edematous and dark red in color.

The wall of the right ventricle was thin and flabby. The left atrium was moderately dilated, but jet lesions were not seen. The lungs were severely congested and oozed blood from cut surfaces (Fig. 3).

**Fig. 4:**
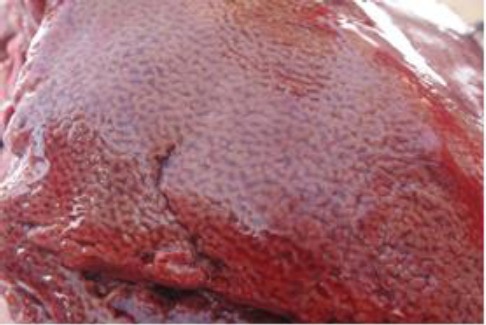
The liver showing typical nutmeg appearance of venous congestion.

The trachea contained bloody froth. The liver was enlarged and showed typical nutmeg appearance (Fig. 4).

**Fig. 5:**
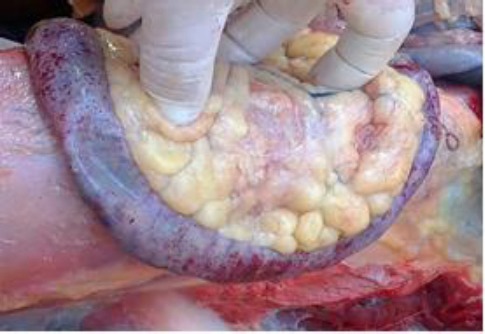
A segment of the small intestine showing severe sub-serosal petichial and ecchymotic hemorrhages.

The kidneys were also swollen and congested. The small intestine contained little food and showed sub-serosal petechial and ecchymotic hemorrhages on a segment measuring about 25 cm (Fig. 5). Marked fat accumulation was noticed on the mesentery and in the abdominal wall while the abdomen contained moderate amount of ascitic fluid.

**Fig. 6:**
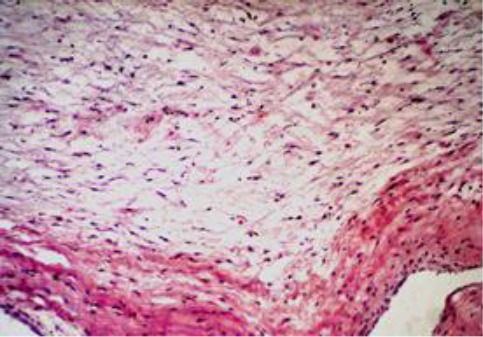
Mitral valve showing fibromyxomatous degeneration formed of loosely arranged stellate fibroblast.(H&E ×10).

**Fig. 7:**
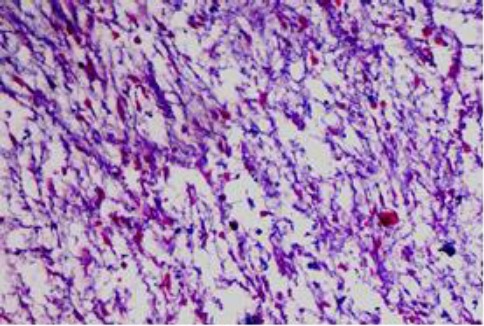
Mitral valve showing fibromyxomatous degeneration and mucopolysaccharide-rich ground substance (Alcian blue, ×10).

On microscopy, the valves showed extensive myxomatous degeneration formed of loosely arranged stellate fibroblast and mucopolysaccharide-rich ground substance (Fig. 6 and 7).

**Fig. 8:**
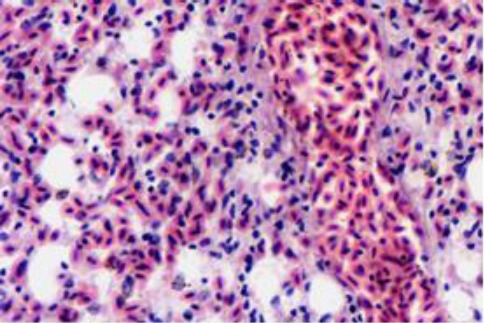
The lung showing edema and severe vascular congestion (H&E, ×40).

**Fig. 9:**
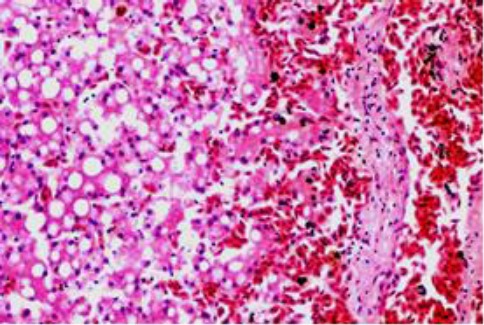
The liver shows congestion in a centrilobular vein and close sinusoids, necrosis in the centrilobular hepatocytes (right) and fatty degeneration in the outer hepatic zones (H&E, ×20).

The pulmonary vessels and capillaries were severely congested with accumulation of transudate in air capillaries (Fig. 8). The hepatic central veins were also severely congested along with the sinusoids of the centrilobular zone. The hepatocytes close to the inner zone were necrosed, while those in the middle and portal zones were loaded with fat vacuoles (Fig. 9).

**Fig. 10:**
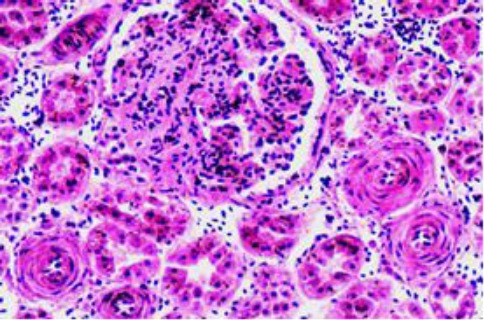
Renal glomeruli are moderately enlarged and show segmental glomerulosclerosis with partial adhesion and narrowing of urinary space. The nearby arterioles are arteriosclerotic.

A considerable number of renal glomeruli showed variable extents of hypercellularity, sclerosis and capsular adhesion. The renal arterioles in many instances had onion like thickening of their walls and narrow lumen (Fig. 10).

**Fig. 11:**
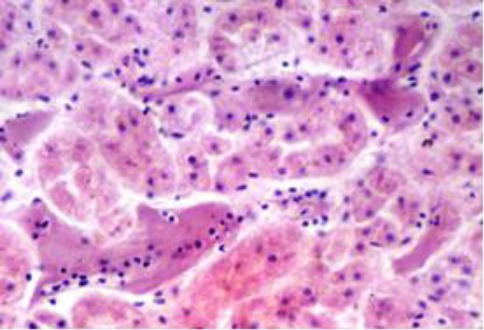
Smooth translucent fibrinous thrombi in renal intertubular capillaries (H&E, ×40).

**Fig. 12:**
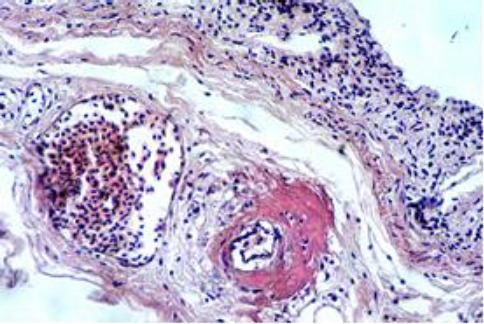
Small intestine showing villous cicatrisation and atrophy. The submucosal arterioles are distorted, partially obliterated and hyalinized (H&E, ×10).

Septic mural thrombi were observed in many medium-sized vessels, while homogenous micro-thrombi were noticed in many capillaries (Fig. 11). The affected segment of the intestine had severe subserosal venous engorgement and hemorrhages. The inter-muscular and sub-mucosal arterioles showed irregularly thickened walls with variable replacement with a homogenous eosinophilic hyaline material replacement and narrowing in their lumen. The overlying mucosal villi were sclerotic and atrophied (Fig. 12).

**Fig. 13:**
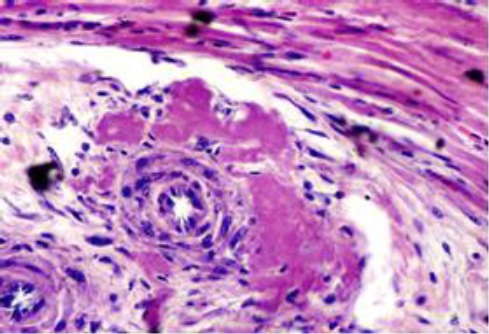
A myocardial arteriole showing wall thickening with hyalinization (H&E, ×40).

Some arterioles of the cardiac muscle have also shown such changes (Fig. 13).

Amyloidosis was ruled out by the negative reaction of Crystal violet.

## Discussion

Necropsy and microscopic examination have illustrated that the most possible cause of death, regardless of other recorded pathological abnormalities, is pulmonary failure. Those abnormalities, however, have certainly participated in deteriorating the general body condition and enhanced death.

The pronounced pulmonary congestion and edema which led to fatal flooding of air capillaries were obviously secondary to cardiac disorder and are probably augmented by the natural sporadic attenuation of the blood - air barrier in ostrich lungs (Maina and Nathaniel, 2001).

Left ventricle hypertrophy and associated pulmonary congestion encountered in this bird can well be explained on the basis of incompetence in the mitral valve. In mitral insufficiency, the back pressure in the left atrium is transmitted to the pulmonary veins and by raising pulmonary arterial pressure causes effects on the right side of the heart including ventricular dilatation (Jubb *et al.*, 2008).

Mitral insufficiency can be a sequel to different valvular abnormalities including endocardiosis. In this case, the gross and microscopic changes detected in the mitral valves were concomitant with those stated for endocardiosis in dogs (Jones *et al.*, 1997; Jubb *et al.*, 2008; Kittleson, 2011; Fox, 2012) and in other animals (Schmidt, 1970; Guarda *et al.*, 1993; Castagnaro *et al.*, 1997; Reef *et al.*, 1998; Sisson *et al.*, 1999; Muller *et al.*, 2010). Some authors (Olkowski *et al.*, 1998; Saif *et al.*, 2008; Schmidt *et al.*, 2008) have reported the occurrence of such condition in different birds with no special reference to ostrich.

The nutmeg appearance of the liver is characteristic of venous congestion, which is a usual sequel to right ventricular failure. The abnormal thickening confined to the arterial vascular walls in the affected intestinal loop is comparable to hyaline arteriosclerosis described by Jones *et al*. (1997) who stated that it is sporadic and of unknown pathogenesis in animals. In this bird, however, we think that it has interfered effectively with normal blood flow and led to ischemic villous cicatrisation and atrophy.

Ischemic changes were not obvious in the cardiac muscle probably because of milder arteriolar involvement. In the kidney, arterial wall thickening was devoid of hyalinization and resembled hyperplastic arteriosclerosis, which was also described by the former authors.

They stated that hyperplastic arteriosclerosis is less encountered in animals and is associated with chronic renal diseases and other disorders associated with hypertension.

Blood flow in those arterioles is certainly diminished due to narrowing in their lumen and this may explain the sclerotic changes in the nearby glomeruli. The existence of micro-thrombi in the renal capillaries is thought to be associated with renal ischemia (Jubb *et al.*, 2008). The septic thrombi noticed also in some renal venules might be due to extension from bacterial pyelitis noticed in some sections.

The vascular changes noticed in the heart, kidney and intestine can be secondary to the back pressures in the vascular system created by the endocardiosis and mitral insufficiency. Available literatures concerning endocardiosis in other animals have not mentioned such changes. The current investigation documents for endocardiosis in ostrich.
